# Etude de la morbidité et de la mortalité chez les patients hospitalisés vaccinés versus les patients hospitalisés non vaccinés contre la COVID-19 dans trois régions du Cameroun

**DOI:** 10.11604/pamj.2024.48.114.40348

**Published:** 2024-07-18

**Authors:** Jean Yves Bevela, Serges Billong, Yacouba Mapoure Njankouo, Ingrid Kenko, Georges Bonsou

**Affiliations:** 1 Département de Santé Publique, Université de Yaoundé I, Yaoundé, Cameroun,; 2 Département de Sciences Cliniques, Université de Douala, Douala, Cameroun,; 3 Département de Biologie Animale, Université de Dschang, Dschang, Cameroun

**Keywords:** COVID-19, vaccination, morbidité et mortalité, Cameroun, COVID-19, vaccination, morbidity and mortality, Cameroon

## Abstract

**Introduction:**

la COVID-19 est une maladie infectieuse et contagieuse déclarée urgence de santé publique de portée internationale en 2020. Compte tenu de la forte morbidité et mortalité, l'une des réponses à cette pandémie est la vaccination, qui a posé un sérieux problème d'acceptation au sein de la population en Afrique subsaharienne (ASS) et au Cameroun en particulier. Ainsi, le but de cette étude était de contribuer à une meilleure réponse à la pandémie au Cameroun en mesurant l'efficacité du vaccin contre la COVID-19. L'objectif de notre travail était de faire une analyse comparative de la morbidité et de la mortalité chez les patients vaccinés atteints de COVID-19 versus les patients non vaccinés atteints de COVID-19, hospitalisés dans les trois régions les plus touchées du Cameroun.

**Méthodes:**

nous avons mené une étude Cas-Témoins avec comme Cas les patients vaccinés contre la COVID-19 et Témoins les patients non vaccinés contre la COVID-19. Nous avons observé la survenue des manifestations cliniques sévères chez des patients vaccinés et non vaccinés atteints de COVID-19 en cours d'hospitalisation pour étudier l'influence de la vaccination sur le devenir de ces patients sur la période allant du 1^er^mai 2021 au 31 mars 2022; dans les unités de prise en charge COVID-19 de l'Hôpital Central de Yaoundé, l'Hôpital Général de Douala, l'Hôpital Laquintinie de Douala et l'Hôpital Régional de Bafoussam.

**Résultats:**

nous avons mené notre étude chez 218 patients hospitalisés COVID-19, soit 109 patients vaccinés et 109 patients non vaccinés dont 51,4% de femmes. L'hypertension artérielle (60,6%) et le diabète (27,5%) étaient plus présents chez les patients non vaccinés. La durée médiane d'hospitalisation était de sept jours pour les patients vaccinés et de cinq jours pour les patients non vaccinés. Le coma (0,7% chez les vaccinés et 79,8% chez les non vaccinés), troubles de la conscience (8,3% chez les vaccinés et 57,8% chez les non vaccinés), céphalées (46,8% chez les vaccinés et 18,3% chez les non vaccinés), pneumonie (78% chez les vaccinés et 78,9% chez les non vaccinés), paludisme (31,2% chez les vaccinés et 19,3% chez les non vaccinés), embolie pulmonaire (14,7% chez les vaccinés et 22% chez les non vaccinés) et maladies thromboemboliques veineuses (1,1% chez les vaccinés et 14,7% chez les non vaccinés) étaient les principales manifestations cliniques sévères. La prévalence de la mortalité était de 1,8% chez les patients vaccinés et de 79,8% chez les patients non vaccinés.

**Conclusion:**

quatre patients vaccinés sur 1000 étaient moins susceptibles de décéder en cours d'hospitalisation par rapport aux patients non vaccinés. Ce qui renforce la place de la vaccination pour le contrôle de l'infection à COVID-19.

## Abstract (en)

**Introduction:**

COVID-19 is an infectious and contagious disease declared as public health emergency of international concern in 2020. Given its high morbidity and mortality, one of the responses to this pandemic is vaccination, which has posed a serious problem of acceptance among the population in sub-Saharan Africa (SSA) and Cameroon in particular. Thus, the purpose of this study was to contribute to a better response to the pandemic in Cameroon by measuring the effectiveness of the COVID-19 vaccine. We carried out a comparative analysis of morbidity and mortality in vaccinated COVID-19 patients versus unvaccinated COVID-19 patients hospitalized in the three most affected regions of Cameroon.

**Methods:**

we conducted a Case-control study with patients vaccinated against COVID-19 as Cases and patients not vaccinated against COVID-19 as controls. We observed the occurrence of severe clinical manifestations in vaccinated and unvaccinated COVID-19 patients during hospitalization to study the influence of vaccination on the outcome of these patients over the period from May 01, 2021, to March 31, 2022; in the COVID management units of Yaoundé Central Hospital, Douala General Hospital, Douala Laquintinie Hospital and Bafoussam Regional Hospital.

**Results:**

we conducted our study in 218 hospitalized COVID-19 patients, 109 vaccinated and 109 unvaccinated patients, 51.4% of whom were women. Arterial hypertension (60.6%) and diabetes (27.5%) were more prevalent in unvaccinated patients. The median length of hospital stay was 07 days for vaccinated patients and 05 days for unvaccinated patients. Coma (0.7% in vaccinated patients and 79.8% in unvaccinated patients), consciousness disorders (8.3% in vaccinated patients and 57.8% in unvaccinated patients), headaches (46.8% in vaccinated patients and 18.3% in unvaccinated patients), pneumonia (78% in vaccinated patients and 78.9% in unvaccinated patients), malaria (31.2% in vaccinated patients and 19.3% in unvaccinated patients), pulmonary embolism (14.7% in vaccinated patients and 22% in unvaccinated patients) and venous thromboembolism (1.1% in vaccinated patients and 14.7% in unvaccinated patients) were the main severe clinical manifestations. The prevalence of mortality was 1.8% in vaccinated patients and 79.8% in unvaccinated patients.

**Conclusion:**

four out of 1000 vaccinated patients were less likely to die during hospitalization compared to unvaccinated patients. This reinforces the importance of vaccination in controlling COVID-19 infection.

## Introduction

La maladie à coronavirus ou coronavirus disease 19 (COVID-19) est une maladie infectieuse émergente causée par le coronavirus SARS-CoV-2 (Severe Acute Respiratory Syndrome coronavirus 2). Très contagieux, la COVID-19 a surpris le monde entier par sa brutalité, sa soudaineté et la rapidité de son expansion [1]. Le 30 janvier 2020 l'Organisation Mondiale de la Santé (OMS) suivant les recommandations du comité d'urgence l'a déclarée urgence de santé publique de portée internationale (USPPI), puis pandémie le 11 mars 2020 [2]. Après la Chine, l'Europe et les Etats Unis d'Amérique, l'Afrique subsaharienne (ASS) connait, depuis mars 2020, l'épidémie à COVID-19. Avant l'arrivée de cette épidémie, l'OMS et les experts en santé prédisaient tous une propagation fulgurante de la COVID-19 en ASS avec une mortalité sans précédent [3]. L'une des réponses face à cette pandémie a été la mise en place de la vaccination anti COVID-19. La vaccination anti COVID-19 depuis son introduction en Afrique Subsaharienne en général et au Cameroun en particulier, pose un sérieux problème d'acceptation. Ce qui a pour conséquence la faible couverture vaccinale anti COVID-19 dans la population camerounaise qui est de 8,8% pour celle qui s'est faite complètement vaccinée et 11,4% pour celle qui a pris une dose de vaccin. Ce qui est bien loin de l'objectif fixé par le gouvernement qui est de faire vacciner au moins 70% de la population camerounaise afin de contenir la pandémie. Une revue de la morbidité et de la mortalité chez les personnes vaccinées versus personnes non vaccinées contre la COVID-19 va du constat fait depuis l'introduction dudit vaccin dans le système de santé du Cameroun en avril 2021, qui montre que malgré la vaccination, certaines personnes font la maladie sous sa forme grave et parfois décèdent. Alors que l'OMS et les pouvoirs publics du Cameroun disent de la vaccination anti COVID-19 que le vaccin limite la morbidité et empêche de faire la forme grave et les complications de la maladie. A l'état actuel des connaissances au Cameroun, il n'existe pas d'études similaires sur l'intérêt de la vaccination anti COVID chez les personnes victime de cette maladie. Ainsi, le but de cette étude était de contribuer à une meilleure riposte à la pandémie COVID-19 au Cameroun.

L'objectif général du travail était d'étudier la morbidité et la mortalité chez les patients vaccinés versus les patients non vaccinés atteints de COVID-19 en cours d'hospitalisation dans les trois régions les plus affectées du Cameroun (Centre, Littoral et Ouest) dans les unités de prise en charge COVID-19. De manière plus spécifique, il s'agissait de comparer les caractéristiques sociodémographiques et cliniques des patients COVID-19 hospitalisés, comparer les comorbidités des patients COVID-19, décrire le profil vaccinal des patients COVID-19 hospitalisés et évaluer l'impact de la vaccination sur le devenir (la durée moyenne d'hospitalisation, la morbidité et de la mortalité) des patients COVID-19 hospitalisés.

## Méthodes

**Conception et type de l'étude:** nous avons mené une étude cas-témoins avec comme cas les patients vaccinés contre la COVID-19 et comme témoins les patients non vaccinés contre la COVID-19. Sur le plan administratif et institutionnel, nous avons rédigé un protocole de recherche et une demande au comité d'éthique de la Faculté de Médecine et des Sciences Biomédicales de l'Université de Yaoundé I afin d'obtenir une clairance éthique. Nous avons soumis au comité d'éthique notre protocole de recherche pour évaluation, commentaires, conseils et approbation avant de commencer nos recherches. Après l'obtention de la clairance éthique N°290/UYI/FMSB/VDRC/CSO du 19 mai 2022, nous avons rédigé des demandes d'autorisation aux responsables des sites choisis afin d'entrer en possession des dossiers médicaux des patients. Après l'obtention des autorisations, nous avons rencontré les responsables des unités de prise en charge COVID-19 qui nous ont donné accès aux dossiers médicaux et registres des patients. Nous avons observé la survenue des manifestations cliniques sévères chez des patients vaccinés et non vaccinés atteints de COVID-19 en cours d'hospitalisation pour étudier l'influence de la vaccination sur le devenir de ces patients.

**Cadre de l'étude:** notre période d'étude a débuté par la rédaction du protocole de recherche durant la période allant d'octobre à décembre 2021 soit une période de trois mois. Durant cette même période, nous avons rédigé des lettres d'autorisation aux directeurs des hôpitaux choisis pour mener nos enquêtes dans les unités de prise en charge COVID-19 choisies. Après autorisation, nous avons débuté la collecte des données dans les différentes unités de prise en charge COVID-19 durant la période allant de janvier à mai 2022 et nous avons choisi de collecter les données dans les dossiers médicaux des patients admis en unité de prise en charge COVID-19 de la période du 1^er^mai 2021 au 31 mars 2022. Notre période d'enregistrement dans la base de données et l'analyse de ces données et les discussions s'est faite de juin à juillet 2022. Durant la période d'août à septembre 2022, nous avons revu les résultats et les différentes analyses que nous avons validés. Notre étude a donc duré 11 mois allant d'octobre 2021 à septembre 2022.

**Lieu de l'étude:** nous avons mené notre étude dans les unités de prise en charge (UPEC) COVID-19 de l'Hôpital Central de Yaoundé, l'Hôpital Général de Douala, l'Hôpital Laquintinie de Douala et l'Hôpital Régional de Bafoussam.

**Participants de l'étude:** nous avons inclus dans l'étude tous les dossiers de patients COVID-19 âgés de plus de 15 ans hospitalisés avec une forme sévère de la maladie, vaccinés ou non, avec un diagnostic de l'infection basé sur un TDR ou PCR antigénique ou un scanner thoracique évocateur. Les paramètres de comparaison entre les patients COVID-19 vaccinés et non vaccinés étaient la durée moyenne d'hospitalisation, la morbidité et la mortalité hospitalière. Le choix des patients vaccinés ou non vaccinés était basé sur l'indicateur « statut vaccinal » dans les dossiers médicaux et/ou registres des patients. Tout dossier ou registre ne comportant pas cet indicateur n'était pas inclus dans l'étude. Nous avons procédé par appariement des « Témoins » avec les « Cas ». La comparaison s'est faite selon les critères d'âge et de sexe. Et chaque témoin devait correspondre à un cas soit pour cette étude 109 Cas et 109 Témoins.

**Variables:** les variables dépendantes étaient des variables à rechercher et à expliquer. Et dans notre étude, il s'agissait de la morbidité et la mortalité chez les patients vaccinés et non vaccinés en cours d'hospitalisation. Les variables indépendantes étaient des variables explicatives. Et dans notre étude, il s'agissait des caractéristiques sociodémographiques et cliniques, des antécédents, les sites de collecte qui étaient les unités de prise en charge de COVID-19 dans les régions choisies, les types de vaccin et le devenir des patients COVID-19 en cours d'hospitalisation.

**Sources de données:** les données ont été collectées dans les dossiers médicaux et les registres des patients à l'aide d'une fiche de collecte de données. Pour chaque témoin « non vacciné » correspondait un cas « vacciné » selon l'âge et le sexe dans chaque unité de prise en charge COVID-19 de chaque formation sanitaire.

**Biais:** nous avons classé les dossiers suivant l'indicateur « statut vaccinal » mentionné dessus mais malheureusement très peu de dossiers avaient cet indicateur mentionné. Le choix des régions a été fait sur la base de la prévalence des Cas de COVID-19 au Cameroun. Et les trois régions les plus touchées étaient le Centre, le Littoral et l'Ouest.

**Sélection de l'échantillon:** nous avons procédé par échantillonnage de convenance des Cas répondant aux critères d'inclusion durant la période de recrutement. Cette technique d'échantillonnage a été choisie pour des raisons pratiques d'accessibilité et de coût plutôt que basé sur une rigueur méthodologique et une volonté d'assurer statistiquement une représentativité.

**Analyse des données:** les données ont été collectées par le logiciel Cs Pro version 7.7 et analysées par le logiciel SPSS version 26. Le traitement de texte a été réalisé sur le logiciel Microsoft Word et les graphiques ont été réalisés avec le logiciel Microsoft Excel. Les variables qualitatives ont été comparées par le test de Khi^2^ tandis que le test T de Student a permis de comparer les variables quantitatives; une valeur de p<0.05 a été considérée comme statistiquement significative.

## Résultats

**Figure 1:**
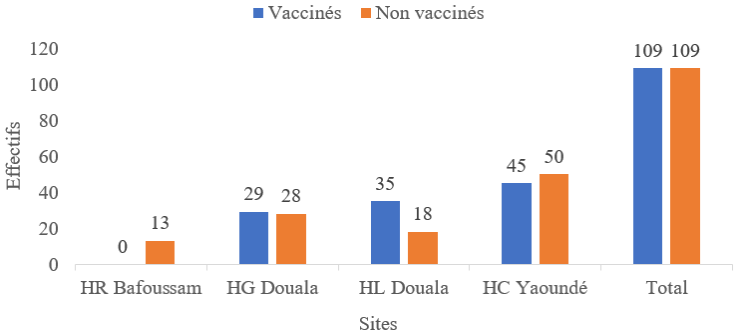
répartition des patients COVID-19 vaccinés et non vaccinés retrouvés dans les unités de prise en charge COVID-19 Hôpital Central de Yaoundé/Hôpital Général de Douala/Hôpital Laquintinie de Douala/Hôpital Régional de Bafoussam, 1^er^ mai 2021 au 31 mars 2022

Au total, nous avons mené l'étude chez 218 patients soit 109 patients vaccinés et 109 patients non vaccinés. Caractéristiques sociodémographiques et cliniques des patients COVID-19 hospitalisés: nous avons mené l'étude dans quatre sites de prise en charge COVID-19, repartis dans les trois régions les plus affectées du Cameroun à savoir le Centre, le Littoral et l'Ouest. A l'Hôpital Régional de Bafoussam (0% vaccinés vs 12% non vaccinés), Hôpital Général de Douala (24,7% vaccinés vs 29,3% non vaccinés), Hôpital Laquintinie de Douala (11% vaccinés vs 18,3% non vaccinés), Hôpital Central de Yaoundé (47,7% vaccinés vs 56,8% non vaccinés) (Figure 1).

**Figure 2:**
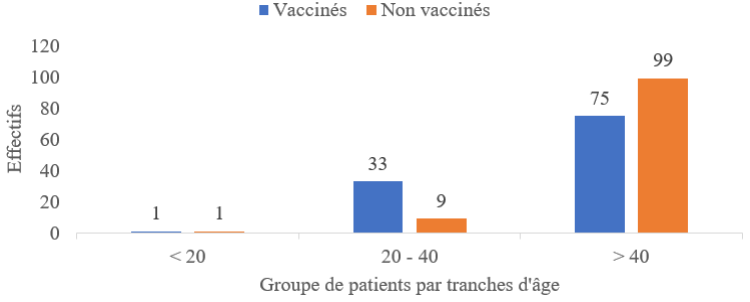
répartition des patients COVID-19 vaccinés et non vaccinés par tranche d'âge, unités de prise en charge COVID-19 Hôpital Central de Yaoundé/Hôpital Général de Douala/Hôpital Laquintinie de Douala/Hôpital Régional de Bafoussam, 1^er^ mai 2021 au 31 mars 2022

La prédominance était de sexe féminin (51,4%), soit un sexe ratio de 1,05. L'âge moyen des patients étaient de 53.9 ± 19.2 ans avec des âges extrêmes compris entre 15 ans et 104 ans. L'âge moyen chez les vaccinés était de 51.8 ± 19.1 ans et chez les non vaccinés était de 56.1 ± 16.6 ans avec P <0,001 (Figure 2). Comorbidité des patients COVID-19: L'HTA représentait la principale comorbidité des patients COVID-19 hospitalisés avec 60,6% chez les non vaccinés vs 39,4% chez les vaccinés. Le diabète représentait 27,5% chez les non vaccinés vs 12,8% chez les vaccinés (Tableau 1). La médiane du nombre de jours d'hospitalisation était de sept jours chez les vaccinés vs cinq jours chez les non vaccinés avec P= 0,029 (Tableau 2).

**Tableau 1:** répartition des comorbidités chez les patients COVID-19 vaccinés et non vaccinés, unités de prise en charge COVID-19 Hôpital Central de Yaoundé/Hôpital Général de Douala/Hôpital Laquintinie de Douala/Hôpital Régional de Bafoussam, 1^er^ mai 2021 au 31 mars 2022

Variables	Total (N = 218)	Vaccinés (Cas) (N = 109)	Non vaccinés (Témoins) (N = 109)
HTA	N=109 (50%)	N=43 (39,4%)	N=66 (60,6%)
Diabète	N=44 (20,2%)	N=14 (12,8%)	N=30 (27,5%)
Asthme	N=2 (0,9%)	N=2 (1,8%)	N=0 (0%)
MRC	N=9 (4,1%)	N=2 (1,8%)	N=7 (6,4%)
IC	N=3 (1,4%)	N=1 (0,9%)	N=2 (1,8%)
HV	N=2 (0,9%)	N=1 (0,9%)	N=1 (0,9%)
VIH	N=8 (3,7%)	N=1 (0,9%)	N=7 (6,4%)
Cancer	N=9 (4,1%)	N=3 (2,8%)	N=6 (5,5%)
Obésité	N=10 (4,6%)	N=6 (5,5%)	N=4 (3,7%)
AVC	N=14 (6,4%)	N=7 (6,4%)	N=7 (6,4%)
Tuberculose	N=2 (0,9%)	N=1 (0,9%)	N=1 (0,9%)
Epilepsie	N=2 (0,9%)	N=1 (0,9%)	N=1 (0,9%)

**HTA:** Hypertension artérielle ; **MRC:** Maladie Rénale Chronique; **IC:** Insuffisance cardiaque; **HV:** Hépatites virales; **VIH:** Virus d'immunodéficience humaine; **AVC:** Accident vasculaire cérébrale

**Tableau 2:** nombre de jours d'hospitalisation chez les patients COVID-19 hospitalisés, vaccinés et non vaccinés, unités de prise en charge COVID-19 Hôpital Central de Yaoundé/Hôpital Général de Douala/Hôpital Laquintinie de Douala/Hôpital Régional de Bafoussam, 1^er^ mai 2021 au 31 mars 2022

Variables	Total (N = 218)	Vaccinés (Cas) (N = 109)	Non vaccinés (Témoins) (N = 109)	Valeur p
**Durée d'hospitalisation (jours)**				
**Médiane (IIQ)**	6,0 (3,0-9,0)	7,0 (4,0-9,0)	5,0 (1,0-9,0)	0,029
**Min-Max**	0-40	0-29	0-40	

IIQ= Intervalle interquartile, dans notre étude la distribution n'étant pas normale, nous avons utilisé la médiane comme paramètre de tendance centrale au lieu de la moyenne car la valeur P< 0,05, donc une médiane (IIQ) comme dans notre étude veut dire 50% des patients COVID 19 avaient une durée d'hospitalisation inférieure à 06 jours et les autres 50% avaient une durée d'hospitalisation supérieure à 06 jours, avec P=0,029 statistiquement significative

Manifestations cliniques sévères des patients COVID-19: la pneumonie était la plus représentée (78% chez les vaccinés vs 78,9% chez les non vaccinés avec P= 0,869), le coma (0,7% chez les vaccinés vs 79,8% chez les non vaccinés avec P< 0,001), troubles de la conscience (8,3% chez les vaccinés vs 57,8% chez les non vaccinés avec P< 0,001), céphalées (46,8% chez les vaccinés vs 18,3% chez les non vaccinés avec P< 0,001), embolie pulmonaire (14,7% chez les vaccinés vs 22% chez les non vaccinés avec P= 0,162), maladie veineuse thrombo-embolique (1,1% chez les vaccinés vs 14,7% chez les non vaccinés avec P= 0,216), insomnie (17,4% chez les vaccinés vs 6,4% chez les non vaccinés avec P= 0;012) (Tableau 3). Profil vaccinal des patients COVID-19: l'Astra Zeneca (20,2%), le Sinopharm (33%), Johnson and Johnson (35,8%) et Pfizer (11%) étaient les vaccins retrouvés dans le profil vaccinal des patients hospitalisés pour COVID-19. Parmi les vaccins multidoses, seulement 39,4% des patients vaccinés ont reçu deux doses de vaccins (Tableau 4). Impact de la vaccination sur le devenir des patients COVID-19: parmi les patients décédés de COVID-19, 1,8% vaccinés vs 79,8% non vaccinés avec P< 0,001 (Tableau 5).

**Tableau 3:** répartition des manifestations cliniques sévères chez les patients COVID-19 hospitalisés, vaccinés et non vaccinés, unités de prise en charge COVID-19 Hôpital Central de Yaoundé/Hôpital Général de Douala/Hôpital Laquintinie de Douala/Hôpital Régional de Bafoussam, 1^er^ mai 2021 au 31 mars 2022

Manifestations Cliniques	Total (N = 218)	Vaccinés (Cas) (N = 109)	Non vaccinés (Témoins) (N = 109)	OR (IC 95%)	Valeur p
**Céphalées**	N=71 (32,6%)	N=51 (46,8%)	N=20 (18,3%)	1,8 (1,4-2,3)	< 0,001
**TC**	N=72 (33,0%)	N=9 (8,3%)	N=63 (57,8%)	0,2 (0,09-0,3)	< 0,001
**Coma**	N=88 (40,4%)	N=1 (0,7%)	N=87 (79,8%)	0,01 (0,002-0,09)	< 0,001
**Hémiplégie**	N=6 (2,8%)	N=2 (1,8%)	N=4 (3,7%)	0,7 (0,2-2,1)	0,683
**Insomnie**	N=26 (11,9%)	N=19 (17,4%)	N=7 (6,4%)	1,6 (1,2-2,1)	0,012
**Pneumonie**	N=171 (78,4%)	N=85 (78,0%)	N=86 (78,9%)	0,9 (0,7-1,3)	0,869
**EP**	N=40 (18,3%)	N=16 (14,7%)	N=24 (22,0%)	0,8 (0,5-1,1)	0,162
**OAP**	N=4 (1,8%)	N=2 (1,8%)	N=2 (1,8%)	1,0 (0,4-2,7)	1,000
**MVTE**	N=39 (17,9%)	N=23 (1,1%)	N=16 (14,7%)	1,2 (0,9-1,7)	0,216
**IU**	N=25 (11,5%)	N=8 (7,3%)	N=17 (15,6%)	0,6 (0,3-1,1)	0,056
**Paludisme**	N=55 (25,2%)	N=34 (31,2%)	N=21 (19,3%)	1,3 (1,03-1,8)	0,043
**Veinite**	N=10 (4,6%)	N=2 (1,8%)	N=8 (7,3%)	0,4 (0,1-1,4)	0,052
**Escarres**	N=5 (2,3%)	N=0 (0%)	N=5 (4,6%)	-	0,060
**MR**	N=34 (15,6%)	N=11 (10,1%)	N=23 (21,1%)	0,6 (0,4-0,9)	0,025

**TC :** Trouble de la conscience; **EP:** Embolie pulmonaire; **OAP:** œdème aigu du poumon; **MVTE:** Maladie veineuse thrombo-embolique; **IU:** Infection urinaire; **MR:** Maladie rénale

**Tableau 4:** répartition du nombre de doses reçues par type de vaccin et par sexe, unités de prise en charge COVID-19 Hôpital Central de Yaoundé/Hôpital Général de Douala/Hôpital Laquintinie de Douala/Hôpital Régional de Bafoussam, 1^er^ mai 2021 au 31 mars 2022

Nombre de doses	SEXE	Astra Zeneca	Johnson and Johnson	Pfizer	Sinopharm	Total général
**1**	Féminin	4	24	8	0	**36**
	Masculin	8	15	2	5	**30**
**Total 1**		**12**	**39**	**10**	**5**	**66**
**2**	Féminin	5	0	0	16	**21**
	Masculin	5	0	2	15	**22**
**Total 2**		**10**	**0**	**2**	**31**	**43**
**Total général**		**22**	**39**	**12**	**36**	**109**

**Tableau 5:** impact de la vaccination sur le devenir des patients COVID-19 hospitalisés, vaccinés et non vaccinés, unités de prise en charge COVID-19 Hôpital Central de Yaoundé/Hôpital Général de Douala/Hôpital Laquintinie de Douala/Hôpital Régional de Bafoussam, 1^er^ mai 2021 au 31 mars 2022

Variables	Total (N = 218)	Vaccinés (Cas) (N = 109)	Non vaccinés (Témoins) (N = 109)	OR (IC à 95%)	Valeur p
**Vivants et guéris**	n=129 (59,2%)	n=107 (98,2%)	n=22 (20,2%)	4,8 (3,3- 7,1)	< 0,001
**Décédés**	n=89 (40,8%)	n=02 (1,8%)	n=87 (79,8%)	0,004 (0,002-0,091)	< 0,001

## Discussion

En résumé, l'étude a été menée chez 218 patients dont 109 vaccinés et 109 non vaccinés. La prédominance était féminine avec un âge moyen de 51 ans. L'HTA et le diabète étaient les principales comorbidités. La pneumonie était la complication de la COVID-19 la plus représentée. Le Johnson and Johnson était le vaccin le plus retrouvée. Les patients vaccinés décédaient moins que les patients non vaccinés. Et les vaccins Astra Zeneca et Pfizer étaient retrouvés chez les patients vaccinés décédés.

**Limites de l'étude:** nous n'avons utilisé que les dossiers des patients ayant l'indicateur « statut vaccinal », notre étude s'est faite uniquement en milieu hospitalier et dans trois régions du Cameroun, nous avons utilisé un échantillonnage de convenance et le coût d'une telle étude dans toutes les régions devait être très conséquent. Ce qui a eu pour effet de limiter nos résultats car avec un échantillon plus représentatif et des investigations dans toutes les régions du Cameroun et pas seulement en milieu hospitalier, nous aurions pu avoir un aperçu complet et global de la place de la vaccination anti COVID-19 au Cameroun.

L'âge moyen des patients vaccinés dans notre étude était de 51,8 ± 19,1 ans comparés aux patients non vaccinés qui était de 56,1 ± 16,6 ans avec une prédominance féminine. Ce qui n'est pas très loin de Jabagi *et al*. [4] en 2021 qui ont trouvé 64,6 ± 6,4 ans chez les patients vaccinés et patients non vaccinés avec une prédominance féminine. Ce qui cadre avec la revue littéraire de la COVID-19 du fait que depuis le début de la pandémie, la population de plus de 50 ans est la plus vulnérable à la COVID-19, l'âge est un facteur de gravité des patients COVID-19 à partir de 50 ans [4] et dans notre contexte, c'est la population la plus réfractaire à la vaccination. La durée médiane d'hospitalisation était de sept jours pour les vaccinés et cinq jours pour les non vaccinés. Ceci peut s'expliquer par le fait que les patients non vaccinés décédaient rapidement en cours d'hospitalisation. Dans notre étude, nous avons un taux de décès à 40,8% (soit 1,8% chez les vaccinés et 79,8% chez les non vaccinés). Ce qui est bien loin de l'étude de Zhu *et al*. [5] qui avait trouvé 86% de décès chez les non vaccinés et 84% chez les vaccinés. L'émergence de la variante Delta est à l'origine de l'augmentation du nombre de Cas de décès contrairement à notre étude où malgré la présence de la variante, nous enregistrons un faible taux de mortalité chez les vaccinés. La pneumonie était à 78% chez les vaccinés et 78,9% chez les non vaccinés. Les maladies veineuses thromboemboliques représentaient 1,1% chez les vaccinés et 14,7% chez les non vaccinés avec un taux élevé de D-dimères à 89,6% (soit 86,5% chez les vaccinés et 93,3% chez les non vaccinés). Ce qui est bien loin de l'étude de Guan *et al*. [6] qui ont retrouvé dans leur étude 59,6% des D-dimères élevées dans les formes graves. Ce résultat montre que dans notre contexte, la durée d'hospitalisation était beaucoup plus longue et les patients étaient beaucoup plus alités avec des facteurs de risque tels que l'âge, les maladies veineuses thromboemboliques, les embolies pulmonaires, la pneumonie, les accidents vasculaires cérébraux, les cancers, les insuffisances cardiaques et rénales.

## Conclusion

Dans notre étude sur la morbidité et la mortalité chez les patients vaccinés vs les patients non vaccinés atteints de COVID-19, un patient vacciné sur trois était moins susceptible de développer une manifestation clinique sévère en cours d'hospitalisation par rapport aux patients non vaccinés. De même, quatre patients vaccinés sur 1000 étaient moins susceptibles de décéder en cours d'hospitalisation que les patients non vaccinés. Cela renforce la place de la vaccination dans le contrôle de l'infection au COVID-19. Cependant, pour une meilleure visibilité, une étude prospective du groupe vacciné et non vacciné devrait être menée dans les ménages, les centres de vaccination internationaux et les unités de prise en charge COVID-19 dans d'autres régions avec une taille d'échantillon considérable afin de généraliser les résultats et de conclure de manière formelle la place et l'efficacité du vaccin COVID-19 dans notre société.

### Etat des connaissances sur le sujet

- *L'OMS et les pouvoirs publics disent de la vaccination anti COVID-19 qu'elle limite la morbidité et empêche le développement des formes graves de la COVID-19;*
- *Il existe un contraste avec la réalité car nous avons des formes graves et des décès post vaccinaux liés à la COVID-19;*
- *Le décès de certains leaders politiques, des voisins et collègues qui ont eu à faire des formes graves de la maladie avait pour conséquence le refus de la vaccination par la population*.

### Contribution de notre étude à la connaissance

- *Cette étude a permis d'avoir les éléments de réponse sur la place actuelle de la vaccination au Cameroun surtout avec la crainte de la population à se faire vacciner contre la COVID-19;*
- *Bien que la vaccination n'empêche d'avoir les manifestations cliniques sévères ou complications de la COVID-19, elle limite tout au moins la mortalité;*
- *Ces résultats peuvent être utilisés comme base de référence pour d'autres études à grande échelle comme des études prospectives et/ou rétrospectives dans les domiciles, les centres de vaccination internationaux et les unités de prise en charge COVID-19 dans les autres régions avec un échantillon considérable afin de généraliser les résultats et de conclure de manière formelle la place et l'efficacité du vaccin contre la COVID-19 au Cameroun*.

## Remerciements

Les auteurs tiennent à remercier les unités de prise en charge COVID-19 de l'Hôpital Central de Yaoundé, Hôpital Général de Douala, Hôpital Laquintinie de Douala et Hôpital Régional de Bafoussam pour avoir facilité le travail de recherche.
